# Approximation properties of *λ*-Kantorovich operators

**DOI:** 10.1186/s13660-018-1795-7

**Published:** 2018-08-02

**Authors:** Ana-Maria Acu, Nesibe Manav, Daniel Florin Sofonea

**Affiliations:** 1 Department of Mathematics and Informatics, Lucian Blaga University of Sibiu, Sibiu, Romania; 2 Department of Mathematics, Science Faculty, Gazi University, Ankara, Turkey

**Keywords:** 41A10, 41A25, 41A36, Kantorovich operators, Bernstein operator, Voronovskaja theorem, Rate of convergence

## Abstract

In the present paper, we study a new type of Bernstein operators depending on the parameter $\lambda\in[-1,1]$. The Kantorovich modification of these sequences of linear positive operators will be considered. A quantitative Voronovskaja type theorem by means of Ditzian–Totik modulus of smoothness is proved. Also, a Grüss–Voronovskaja type theorem for *λ*-Kantorovich operators is provided. Some numerical examples which show the relevance of the results are given.

## Introduction

In 1912, Bernstein [10] defined the Bernstein polynomials in order to prove Weierstrass’s fundamental theorem. The Bernstein polynomials have many notable approximation properties, which made them an area of intensive research. For more details on this topic, we can refer the readers to excellent monographs [17] and [16]. The Bernstein operators are given by

1 $$ B_{n}:C[0,1]\to C[0,1],\qquad B_{n}(f;x)= \sum _{k=0}^{n} f \biggl(\frac{k}{n} \biggr)b_{n,k}(x), $$

where

$$b_{n,k}(x)={n \choose k}x^{k}(1-x)^{n-k},\quad x\in[0,1]. $$

Very recently, Cai et al. [11] introduced and considered a new generalization of Bernstein polynomials depending on the parameter *λ* as follows:

2 $$ B_{n,\lambda}(f;x)= \sum_{k=0}^{n} \tilde {b}_{n,k}(\lambda;x)f \biggl(\frac{k }{n} \biggr), $$

where $\lambda\in[-1,1]$ and $\tilde{b}_{n,k},k=0,1,\dots$, are defined below:

$$\begin{aligned} &\tilde{b}_{n,0}(\lambda;x)=b_{n,0}(x)- \frac{\lambda }{n+1}b_{n+1,1}(x), \\ &\tilde{b}_{n,k}(\lambda;x)=b_{n,k}(x)+\lambda \biggl( \frac {n-2k+1}{n^{2}-1}b_{n+1,k}(x)-\frac{n-2k-1}{n^{2}-1}b_{n+1,k+1}(x) \biggr), \\ &\tilde{b}_{n,n}(\lambda;x)=b_{n,n}(x)- \frac{\lambda}{n+1}b_{n+1,n}(x). \end{aligned}$$

In the particular case, when $\lambda=0$, *λ*-Bernstein operators reduce to the well-known Bernstein operators. The authors of [11] have deeply studied many approximation properties of *λ*-Bernstein operators such as uniform convergence, rate of convergence in terms of modulus of continuity, Voronovskaja type pointwise convergence, and shape preserving properties.

The classical Kantorovich operators are the integral modification of Bernstein operators so as to approximate Riemann integrable functions defined on the interval $[0,1]$. These operators were introduced by Kantorovich [18] and attracted the interest of and were studied by a number of authors. Özarslan and Duman [19] considered modified Kantorovich operators and showed that the order of approximation to a function by these operators is at least as good as that of the ones classically used. Dhamija and Deo [13] introduced a King type modification of Kantorovich operators and proved that the error estimation of these operators is better than that of the classical operators. Inequalities for the Kantorovich type operators in terms of moduli of continuity were studied in [6]. In the last years, transferring of approximation by linear positive operators to the q-calculus has been an active area of research. We mention here the papers [3, 5, 7, 9, 12] where *q*-analogue of Kantorovich type operators was introduced and convergence theorems and Voronovskaja type results were proved. Our aim of this paper is to study approximation properties and asymptotic type results concerning the Kantorovich variant of *λ*-Bernstein operators, namely

3 $$ K_{n,\lambda}(f;x)=(n+1) \sum_{k=0}^{n} \tilde{b}_{n,k}(x) \int_{\frac {k}{n+1}}^{\frac{k+1}{n+1}}f(t)\,dt. $$

## Preliminary results

In this section by direct computation we give the moments of the *λ*-Kantorovich operators. Also, the central moments and upper bounds of them are calculated.

### Lemma 2.1

*The* *λ*-*Kantorovich operators verify*

(i) $K_{n,\lambda}(e_{0};x)=1$;
(ii) $K_{n,\lambda}(e_{1};x)= x+\frac{1}{2}\cdot\frac {1-2x}{n+1}+\frac{1-2x+x^{n+1}-(1-x)^{n+1}}{n^{2}-1}\lambda$;
(iii) $K_{n,\lambda}(e_{2};x)=x^{2}- \frac{1}{3}\cdot\frac {9nx^{2}-6nx+3x^{2}-1}{(n+1)^{2}}+\frac {2(-2x^{2}n+x^{n+1}n+xn+x^{n+1}-x)\lambda}{(n-1)(n+1)^{2}}$;
(iv) $K_{n,\lambda}(e_{3};x)=x^{3}- \frac {24n^{2}x^{3}-18n^{2}x^{2}+4nx^{3}+18nx^{2}+4x^{3}-14nx-1}{4(n+1)^{3}}+\frac{\lambda }{2(n+1)^{3}(n-1)}\cdot (-12n^{2}x^{3} + 6n^{2}x^{2} + 12x^{3}n + 6x^{n+1}n^{2} - 30x^{2}n + 12x^{n+1}n + 6xn + 7x^{n+1} - (1 - x)^{n+1} - 8x + 1 )$;
(v) $K_{n,\lambda}(e_{4};x)= \frac{1}{5(n+1)^{4}} \{ 5n^{5}x^{4}-30n^{3}x^{4}+40n^{3}x^{3}+55n^{2}x^{4}-120n^{2}x^{3}-30nx^{4}+75n^{2}x^{2} +80nx^{3}-75nx^{2}+30nx+1 \}+ \frac{2\lambda }{(n-1)(n+1)^{4}} \{-4n^{3}x^{4}+2n^{3}x^{3}+12n^{2}x^{4}-24n^{2}x^{3}-8x^{4}n +2x^{n+1}n^{3}+6n^{2}x^{2}+22x^{3}n+6x^{n+1}n^{2}-24x^{2}n+7x^{n+1}n+3xn+3x^{n+1}-3x \}$.

### Lemma 2.2

*The central moments of* *λ*-*Kantorovich operators are given below*:

(i) $K_{n,\lambda}(t-x;x)= \frac{1-2x}{2(n+1)}+\frac{\lambda (1-2x+x^{n+1}-(1-x)^{n+1})}{n^{2}-1}$;
(ii) $K_{n,\lambda}((t-x)^{2};x)= \frac {3x(1-x)(n-1)+1}{3(n+1)^{2}}+\frac{2\lambda x(1-x)}{(n-1)(n+1)^{2}} \{ [(1-x)^{n}+x^{n} ](n+1)-2 \}$.

### Lemma 2.3

*The central moments of* *λ*-*Kantorovich operators verify*

$$\bigl\vert K_{n,\lambda}(t-x;x) \bigr\vert \leq\mu(n,\lambda)\quad\textit{and}\quad \bigl\vert K_{n,\lambda}\bigl((t-x)^{2};x\bigr) \bigr\vert \leq \nu(n,\lambda), $$

*where* $\mu(n,\lambda)= \frac{1}{2(n+1)}+\frac{|\lambda|}{n^{2}-1}$ *and* $\nu(n,\lambda)= \frac{3n+4}{12(n+1)^{2}}+\frac{|\lambda |}{2(n^{2}-1)}$ *for* $n>2$.

### Lemma 2.4

*The* *λ*-*Kantorovich operators verify*:

(i) $\lim_{n\to\infty}nK_{n,\lambda} (t-x;x )=\frac{1-2x}{2}$;
(ii) $\lim_{n\to\infty}nK_{n,\lambda} ((t-x)^{2};x )=x(1-x)$;
(iii) $\lim_{n\to\infty}n^{2}K_{n,\lambda} ((t-x)^{4};x )=3x^{2}(1-x)^{2}$;
(iv) $\lim_{n\to\infty}n^{3}K_{n,\lambda} ((t-x)^{6};x )=15x^{3}(1-x)^{3}$.

## Convergence properties of $K_{n,\lambda}$

In this section we investigate the approximation properties of these operators, and we estimate the rate of convergence by using moduli of continuity.

### Theorem 3.1

*If* $f\in C[0,1]$, *then*

$$\lim_{n\to\infty}K_{n,\lambda}(f;x)=f(x)\quad\textit{uniformly on } [0,1]. $$

### Proof

Using Lemma 2.1 gives that

$$\lim_{n\to\infty}K_{n,\lambda}(e_{k};x)=e_{k}(x)\quad \text{uniformly on } [0,1] \text{ for } k\in\{0,1,2\}. $$

Applying the Bohmann–Korovkin theorem, we get the result. □

### Theorem 3.2

*If* $g\in C[0,1]$, *then*

$$\bigl\vert K_{n,\lambda}(g;x)-g(x) \bigr\vert \leq 2 \omega\bigl(g;\sqrt{ \nu(n;\lambda)}\bigr), $$

*where* *ω* *is the usual modulus of continuity*.

### Proof

Using the following property of modulus of continuity

$$\bigl\vert g(t)-g(x) \bigr\vert \leq\omega(g; \delta) \biggl( \frac{(t-x)^{2}}{\delta^{2}}+1 \biggr), $$

we obtain

$$\begin{aligned} \bigl\vert K_{n,\lambda}(g;x)-g(x) \bigr\vert \leq K_{n,\lambda}\bigl( \bigl\vert g(t)-g(x) \bigr\vert ;x\bigr) \leq\omega(g; \delta) \biggl(1+ \dfrac{1}{\delta^{2}}K_{n,\lambda }\bigl((t-x)^{2};x\bigr) \biggr). \end{aligned}$$

So, if we choose $\delta=\sqrt{\nu(n;\lambda)}$, we have the desired result. □

### Theorem 3.3

*If* $g\in C^{1}[0,1]$, *then*

$$\bigl\vert K_{n,\lambda}(g;x)-g(x) \bigr\vert \leq \mu(n;\lambda) \bigl\vert g^{\prime}(x) \bigr\vert +2 \sqrt{\nu(n;\lambda)}\omega \bigl(g^{\prime},\sqrt{\nu(n;\lambda)}\bigr). $$

### Proof

Let $g\in C^{1}[0,1]$. For any $x, t\in[0,1]$, we have

$$g(t)-g(x)=g^{\prime}(x) (t-x)+ \int_{x}^{t} \bigl(g^{\prime }(y)-g^{\prime}(x) \bigr)\,dy, $$

so we get

$$K_{n,\lambda} \bigl(g(t)-g(x);x \bigr)=g^{\prime}(x)K_{n,\lambda}(t-x;x) +K_{n,\lambda} \biggl( \int_{x}^{t}\bigl(g^{\prime}(y)-g^{\prime }(x) \bigr)\,dy;x \biggr). $$

Using the following well-known property of modulus of continuity

$$\bigl\vert g(y)-g(x) \bigr\vert \leq\omega(g;\delta) \biggl( \frac{ \vert y-x \vert }{\delta }+1 \biggr), \quad \delta>0, $$

we have

$$\biggl\vert \int_{x}^{t} \bigl\vert g^{\prime}(y)-g^{\prime}(x) \bigr\vert \,dy \biggr\vert \leq \omega\bigl(g^{\prime};\delta\bigr) \biggl[ \frac{(t-x)^{2}}{\delta}+ \vert t-x \vert \biggr]. $$

Therefore,

$$\begin{aligned} \bigl\vert K_{n,\lambda}(g;x)-g(x) \bigr\vert \leq{}& \bigl\vert g^{\prime}(x) \bigr\vert \cdot \bigl\vert K_{n,\lambda }(t-x;x) \bigr\vert \\ &{} + \omega\bigl(g^{\prime};\delta\bigr) \biggl\{ \frac{1}{\delta}K_{n,\lambda } \bigl((t-x)^{2};x \bigr) + K_{n,\lambda}\bigl( \vert t-x \vert ;x\bigr) \biggr\} . \end{aligned}$$

Using the Cauchy–Schwarz inequality, we obtain

$$\begin{aligned} \bigl\vert K_{n,\lambda}(g;x)-g(x) \bigr\vert \leq{}& \bigl\vert g^{\prime}(x) \bigr\vert \bigl\vert K_{n,\lambda}(t-x;x) \bigr\vert \\ &{}+ \omega\bigl(g^{\prime},\delta\bigr) \biggl\{ \frac{1}{\delta} \sqrt{K_{n,\lambda} \bigl((t-x)^{2};x \bigr)}+1 \biggr\} \sqrt{K_{n,\lambda} \bigl((t-x)^{2};x \bigr)} \\ \leq{}& \bigl\vert g^{\prime}(x) \bigr\vert \mu(n;\lambda)+\omega \bigl(g^{\prime},\delta\bigr) \cdot \biggl\{ \frac{1}{\delta}\sqrt{\nu(n; \lambda)}+1 \biggr\} \sqrt {\nu(n;\lambda)}. \end{aligned}$$

Choosing $\delta=\sqrt{\nu(n;\lambda)}$, we find the desired inequality. □

In order to give the next result, we recall the definition of *K*-functional:

$$K_{2}(g,\delta):=\inf \bigl\{ \Vert g-h \Vert + \delta \bigl\Vert h^{\prime\prime} \bigr\Vert :h\in W^{2}[0,1] \bigr\} , $$

where

$$W^{2}[0,1]= \bigl\{ h \in C[0,1]: h^{\prime\prime} \in C[0,1] \bigr\} , $$

$\delta\geq0$ and $\Vert \cdot \Vert $ is the uniform norm on $C[0,1]$. The second order modulus of continuity is defined as follows:

$$\omega_{2} (g, \sqrt{\delta} ) = \sup_{0 < h \leq\sqrt {\delta}}\sup _{x,x+2h \in[0,1]} \bigl\{ \bigl\vert g(x+2h)-2g(x+h)+g(x) \bigr\vert \bigr\} . $$

It is well known that *K*-functional and the second order modulus of continuity $\omega_{2} (g, \sqrt{\delta} )$ are equivalent, namely

4 $$ K_{2}(g,\delta) \leq C \omega_{2} (g, \sqrt{ \delta} ), $$

where $\delta\geq0$ and $C>0$.

### Theorem 3.4

*If* $g \in C[0,1]$, *then*

$$\bigl\vert K_{n,\lambda}(g;x)-g(x) \bigr\vert \leq C \omega_{2} \biggl(g, \frac {1}{2}\sqrt{ \nu(n;\lambda)+\mu^{2}(n,\lambda)} \biggr)+\omega \bigl(g,\mu(n;\lambda) \bigr), $$

*where* *C* *is a positive constant*.

### Proof

Denote $\varepsilon_{n,\lambda}(x)= x+\frac{1}{2}\cdot \frac{1-2x}{n+1}+\frac{1-2x+x^{n+1}-(1-x)^{n+1}}{n^{2}-1}\lambda $ and

5 $$ \tilde{K}_{n,\lambda}(g;x)=K_{n,\lambda}(g;x)+g(x)- g\bigl( \varepsilon_{n,k}(x)\bigr). $$

It follows immediately

$$\begin{aligned} &\tilde{K}_{n,\lambda}(e_{0};x)= {K}_{n,\lambda}(e_{0};x)=1, \qquad \tilde{K}_{n,\lambda}(e_{1};x)= K_{n,\lambda}(e_{1};x)+x- \varepsilon _{n,\lambda}(x)=x. \end{aligned}$$

Applying $\tilde{K}_{n,\lambda}$ to Taylor’s formula, we get

$$\tilde{K}_{n,\lambda}(h;x)=h(x)+\tilde{K}_{n,\lambda} \biggl( \int _{x}^{t}(t-y)h^{\prime\prime}(y)\,dy;x \biggr). $$

Therefore

$$\tilde{K}_{n,\lambda}(h;x) = h(x)+{K}_{n,\lambda} \biggl( \int _{x}^{t}(t-y)h^{\prime\prime}(y)\,dy;x \biggr)- \int_{x}^{\varepsilon _{n,k}(x)} \bigl(\varepsilon_{n,k}(x)-y \bigr)h^{\prime\prime}(y)\,dy. $$

This implies that

$$\begin{aligned} \bigl\vert \tilde{K}_{n,\lambda}(h;x)-h(x) \bigr\vert & \leq \biggl\vert {K}_{n,\lambda} \biggl( \int_{x}^{t}(t-y)h^{\prime\prime}(y)\,dy;x \biggr) \biggr\vert + \biggl\vert \int_{x}^{\varepsilon_{n,\lambda}(x)} \bigl(\varepsilon _{n,\lambda}(x)-y \bigr)h^{\prime\prime}(y)\,dy \biggr\vert \\ & \leq{K}_{n,\lambda}\bigl((t-x)^{2};x\bigr) \bigl\Vert h^{\prime\prime} \bigr\Vert + \bigl(\varepsilon_{n,\lambda}(x)-x \bigr)^{2} \bigl\Vert h^{\prime \prime} \bigr\Vert \\ &\leq \bigl[\nu(n; \lambda)+\mu^{2}(n;\lambda) \bigr] \bigl\Vert h^{\prime\prime} \bigr\Vert . \end{aligned}$$

In view of (5) we obtain

6 $$ \bigl\vert \tilde{K}_{n,\lambda}(g;x) \bigr\vert \leq \bigl\vert {K}_{n,\lambda}(g;x) \bigr\vert + \bigl\vert g(x) \bigr\vert + \bigl\vert g\bigl(\varepsilon_{n,\lambda}(x)\bigr) \bigr\vert \leq3 \Vert g \Vert . $$

Now, for $g \in C[0,1]$ and $h \in W^{2}[0,1]$, using (5) and (6) we get

$$\begin{aligned} & \bigl\vert {K}_{n,\lambda}(g;x)-g(x) \bigr\vert \\ &\quad= \bigl\vert \tilde{K}_{n,\lambda }(g;x)-g(x)+g\bigl(\varepsilon_{n,\lambda}(x)\bigr)-g(x) \bigr\vert \\ & \quad\leq \bigl\vert \tilde{K}_{n,\lambda}(g-h;x) \bigr\vert + \bigl\vert \tilde {K}_{n,\lambda}(h;x)-h(x) \bigr\vert + \bigl\vert h(x)-g(x) \bigr\vert + \bigl\vert g\bigl(\varepsilon_{n,\lambda }(x)\bigr)-g(x) \bigr\vert \\ &\quad \leq4 \Vert g-h \Vert + \bigl[\nu(n,\lambda)+\mu^{2}(n,\lambda ) \bigr] \bigl\Vert h^{\prime\prime} \bigr\Vert + \omega \bigl(g,\mu(n,\lambda) \bigr). \end{aligned}$$

Taking the infimum on the right-hand side over all $h \in W^{2}[0,1]$, we have

$$\begin{aligned} & \bigl\vert {K}_{n,\lambda}(g;x)-g(x) \bigr\vert \leq4K_{2} \biggl(g,\frac{1}{4} \bigl(\nu (n,\lambda)+\mu^{2}(n,\lambda) \bigr) \biggr) + \omega \bigl(g,\mu(n;\lambda) \bigr). \end{aligned}$$

Finally, using the equivalence between *K*-functional and the second order modulus of continuity (4), the proof is completed. □

## Voronovskaja type theorems

In the following we prove a quantitative Voronovskaja type theorem for the operator $K_{n,\lambda}$ by means of the Ditzian–Totik modulus of smoothness defined as follows:

7 $$ \omega_{\phi}(g;t)=\sup_{0< h\leq t} \biggl\{ \biggl\vert g \biggl(x+\frac{h\phi(x)}{2} \biggr)-g \biggl(x-\frac{h\phi(x)}{2} \biggr) \biggr\vert ,x\pm\frac{h\phi(x)}{2}\in[ 0,1] \biggr\} , $$

where $\phi(x) =\sqrt{x(1-x)}$ and $g\in C[0,1]$. The corresponding *K*-functional of the Ditzian–Totik first order modulus of smoothness is given by

8 $$\begin{aligned} {K}_{\phi}(g;t)=\inf_{h\in W_{\phi}[0,1]} \bigl\{ \bigl\vert \vert g-h \vert \bigr\vert +t \bigl\vert \bigl\lvert \phi h^{\prime } \bigr\vert \bigr\vert \bigr\} \quad (t>0), \end{aligned}$$

where $W_{\phi}[0,1]=\{h:h\in AC_{\mathrm{loc}}[0,1],\|\phi h^{\prime}\| <\infty\}$ and $AC_{\mathrm{loc}}[0,1]$ is the class of absolutely continuous functions on every interval $[a,b]\subset[0,1]$. Between *K*-functional and the Ditzian–Totik first order modulus of smoothness, there is the following relation:

9 $$\begin{aligned} {K}_{\phi}(g;t)\leq C\omega_{\phi}(g;t), \end{aligned}$$

where $C>0$ is a constant.

### Theorem 4.1

*For any* $g\in C^{2}[0,1]$ *and* *n* *sufficiently large*, *the following inequality holds*:

$$\bigl\vert K_{n,\lambda}(g;x)-g(x)-A_{n}(x;\lambda)g^{\prime }(x)-B_{n}(x; \lambda)g^{\prime\prime}(x) \bigr\vert \leq \frac{1}{n} C \phi^{2}(x)\omega_{\phi} \bigl(g^{\prime\prime},n^{-1/2} \bigr), $$

*where*

$$\begin{aligned} &A_{n}(x;\lambda)= \frac{(1-2x)(n-1+2\lambda)}{2(n^{2}-1)}+\lambda\frac {x^{n+1}-(1-x)^{n+1}}{n^{2}-1}; \\ & B_{n}(x;\lambda)= \frac{3x(1-x)(n-1)+1}{6(n+1)^{2}}+\frac{\lambda x(1-x)}{(n-1)(n+1)^{2}} \bigl\{ \bigl[(1-x)^{n}+x^{n} \bigr](n+1)-2 \bigr\} \end{aligned}$$

*and* *C* *is a positive constant*.

### Proof

For $g\in C^{2}[0,1], t,x\in[0,1]$, by Taylor’s expansion, we have

$$g(t)-g(x)=(t-x)g^{\prime}(x)+ \int_{x}^{t}(t-y)g^{\prime\prime}(y)\,dy. $$

Hence

$$\begin{aligned} g(t)-g(x)-(t-x)g^{\prime}(x)- \frac {1}{2}(t-x)^{2}g^{\prime\prime}(x) &= \int_{x}^{t}(t-y)g^{\prime\prime}(y)\,dy- \int_{x}^{t}(t-y)g^{\prime \prime}(x)\,dy \\ &= \int_{x}^{t}(t-y)\bigl[g^{\prime\prime}(y)-g^{\prime\prime}(x) \bigr]\,dy. \end{aligned}$$

Applying $K_{n,\lambda}(\cdot;x)$ to both sides of the above relation, we get

10 $$\begin{aligned} & \bigl\vert K_{n,\lambda}(g;x)-g(x)-A_{n}(x; \lambda)g^{\prime }(x)-B_{n}(x;\lambda)g^{\prime\prime}(x) \bigr\vert \\ &\quad\leq K_{n,\lambda} \biggl( \biggl\vert \int_{x}^{t} \vert t-y \vert \biggr\vert g^{\prime\prime }(y)-g^{\prime\prime}(x) \vert \,dy \vert ;x \biggr). \end{aligned}$$

The quantity $\vert \int_{x}^{t} \vert g^{\prime\prime}(y)-g^{\prime \prime}(x) \vert |t-y|\,dy \vert $ was estimated in [15, p. 337] as follows:

11 $$ \biggl\vert \int_{x}^{t} \bigl\vert g^{\prime\prime}(y)-g^{\prime\prime }(x) \bigr\vert \vert t-y \vert \,dy \biggr\vert \leq2 \bigl\Vert g^{\prime\prime}-h \bigr\Vert (t-x)^{2}+2 \bigl\Vert \phi h^{\prime} \bigr\Vert \phi^{-1}(x) \vert t-x \vert ^{3}, $$

where $h\in W_{\phi}[0,1]$.

Using Lemma 2.4 it follows that there exists a constant $C>0$ such that, for *n* sufficiently large,

12 $$ K_{n,\lambda} \bigl((t-x)^{2};x \bigr)\leq \frac{C}{2n}\phi^{2}(x)\quad\text{and}\quad K_{n,\lambda} \bigl((t-x)^{4};x \bigr)\leq\frac{C}{2n^{2}}\phi^{4}(x). $$

From (10)–(12) and applying the Cauchy–Schwarz inequality, we get

$$\begin{aligned} & \bigl\vert K_{n,\lambda}(g;x)-g(x)-A_{n}(x;\lambda)g^{\prime }(x)-B_{n}(x; \lambda)g^{\prime\prime}(x) \bigr\vert \\ & \quad\leq2 \bigl\Vert g^{\prime\prime}-h \bigr\Vert K_{n,\lambda} \bigl((t-x)^{2};x \bigr)+2 \bigl\Vert \phi h^{\prime} \bigr\Vert \phi^{-1}(x)K_{n,\lambda} \bigl( \vert t-x \vert ^{3};x \bigr) \\ &\quad\leq \frac{C}{n}\phi^{2}(x) \bigl\Vert g^{\prime\prime}-h \bigr\Vert +2 \bigl\Vert \phi h^{\prime } \bigr\Vert \phi^{-1}(x) \bigl\{ K_{n,\lambda}(t-x)^{2};x \bigr\} ^{1/2} \bigl\{ K_{n,\lambda} \bigl((t-x)^{4};x \bigr) \bigr\} ^{1/2} \\ &\quad\leq \frac{C}{n}\phi^{2}(x) \bigl\Vert g^{\prime\prime}-h \bigr\Vert +\phi^{2}(x)\frac {C}{n\sqrt{n}} \bigl\Vert \phi h^{\prime} \bigr\Vert \leq\frac{C}{n}\phi^{2}(x) \bigl\{ \bigl\Vert g^{\prime\prime}-h \bigr\Vert +n^{-1/2} \bigl\Vert \phi h^{\prime} \bigr\Vert \bigr\} . \end{aligned}$$

Taking the infimum on the right-hand side of the above relations over $h\in W_{\phi}[0,1]$, the theorem is proved. □

### Corollary 4.1

*If* $g\in C^{2}[0,1]$, *then*

$$\lim_{n\to\infty}n \bigl\{ K_{n,\lambda}(g;x)-g(x)-A_{n}(x; \lambda )g^{\prime}(x)-B_{n}(x;\lambda)g^{\prime\prime}(x) \bigr\} =0, $$

*where* $A_{n}(x;\lambda)$ *and* $B_{n}(x;\lambda)$ *are defined in Theorem *4.1.

Using the least concave majorant of the modulus of continuity, a Grüss inequality for the positive linear operators was obtained in [4]. This result generated a great deal of interest after its publication. Acar et al. [2] gave a Grüss type approximation theorem and a Grüss–Voronovskaja type theorem for a class of sequences of linear positive operators. A significant contribution in this direction has been made by many authors, we refer the readers to [1, 8, 14, 20].

Next, we will provide a Grüss–Voronovskaja type theorem for *λ*-Kantorovich operators.

### Theorem 4.2

*Let* $f, g\in C^{2}[0,1]$. *Then*, *for each* $x\in[0,1]$,

$$\begin{aligned} \lim_{n\to\infty} n \bigl\{ K_{n,\lambda}\bigl((fg);x \bigr)-K_{n,\lambda }(f;x)K_{n,\lambda}(g;x) \bigr\} = f'(x)g'(x) x(1-x). \end{aligned}$$

### Proof

The following relation holds:

$$\begin{aligned} &K_{n,\lambda}\bigl((fg);x\bigr)- K_{n,\lambda}(f;x)K_{n,\lambda}(g;x) \\ &\quad= K_{n,\lambda}\bigl((fg);x\bigr) - f(x)g(x) - (fg)'(x)A_{n}(x; \lambda) - {(fg)^{\prime\prime}(x)} B_{n}(x;\lambda) \\ &\qquad{} - g(x) \bigl\{ K_{n,\lambda}(f;x) - f(x) - f'(x)A_{n}(x; \lambda) - {f''(x)}B_{n}(x;\lambda) \bigr\} \\ &\qquad{} - K_{n,\lambda}(f;x) \bigl\{ K_{n,\lambda}(g;x) - g(x) - g'(x)A_{n}(x;\lambda) - {g''(x)}B_{n}(x; \lambda) \bigr\} \\ & \qquad{}+ B_{n}(x;\lambda) \bigl\{ f(x)g''(x)+2f'(x)g'(x) - g''(x)K_{n,\lambda }(f;x) \bigr\} \\ &\qquad{}+ A_{n}(x;\lambda) \bigl\{ f(x)g'(x) - g'(x)K_{n,\lambda}(f;x) \bigr\} . \end{aligned}$$

Now, by using Theorem 3.1 and Corollary 4.1, we get

$$\begin{aligned} &\lim_{n\rightarrow\infty} n \bigl\{ K_{n,\lambda }\bigl((fg);x \bigr)-K_{n,\lambda}(f;x)K_{n,\lambda}(g;x) \bigr\} \\ &\quad= \lim_{n\rightarrow\infty} 2n f'(x)g'(x)B_{n}(x; \lambda) + \lim_{n\rightarrow\infty} n{g''(x)} \bigl\{ f(x)-K_{n,\lambda }(f;x) \bigr\} B_{n}(x;\lambda) \\ &\qquad{}+ \lim_{n\rightarrow\infty} n{g'(x)} \bigl\{ f(x)-K_{n,\lambda }(f;x) \bigr\} A_{n}(x;\lambda) = f'(x)g'(x) x(1-x). \end{aligned}$$

 □

## Numerical results

In this section we will analyze the theoretical results presented in the previous sections by numerical examples.

### Example 1

**Figure 1:**
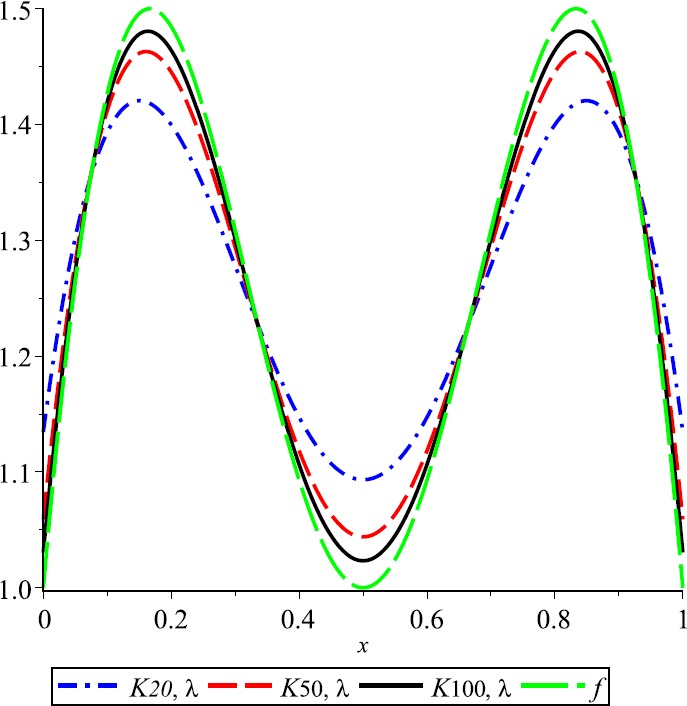
Approximation process

**Figure 2:**
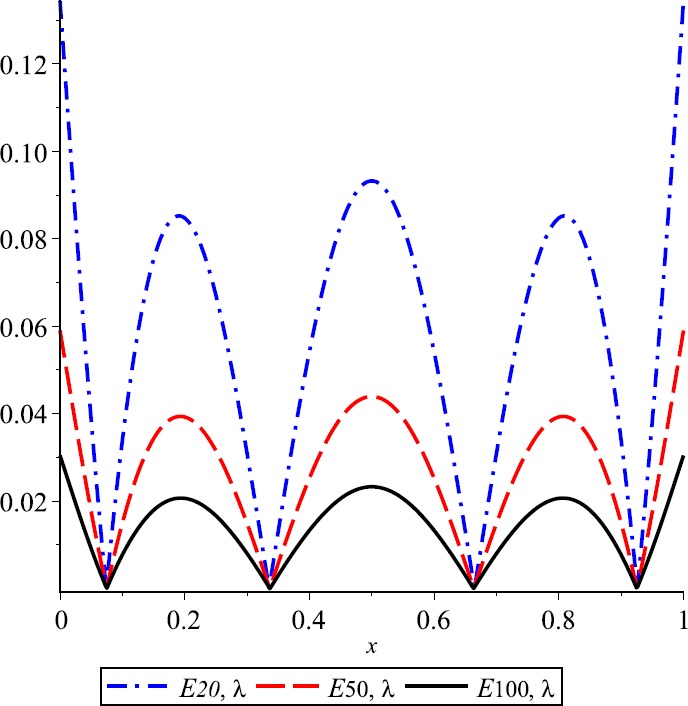
Error of approximation

Let $\lambda=0.3$, $f(x)= \cos(2\pi x)+2\operatorname{sin}(\pi x)$ and $E_{n,\lambda}(f;x)= \vert f(x)-K_{n,\lambda}(f;x) \vert $ be the error function of *λ*-Kantorovich operators. In Fig. 1 the graphs of function f and operator $K_{n,\lambda}$ for $n=20$, $n=50$, and $n=100$ are given, respectively. This example explains the convergence of the operators $K_{n,\lambda}$ that are going to the function f if the values of n are increasing. Also, the error of approximation is illustrated in Fig. 2.

### Example 2

**Figure 3:**
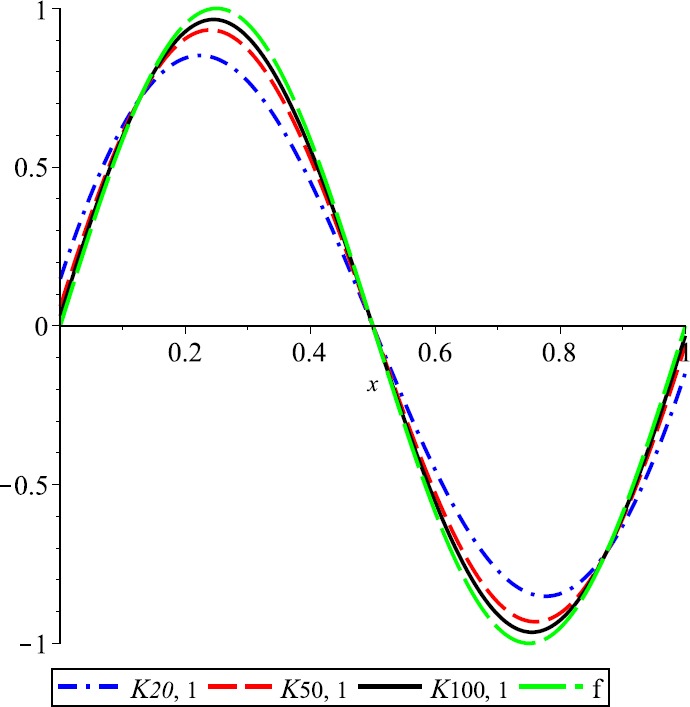
Approximation process

**Figure 4:**
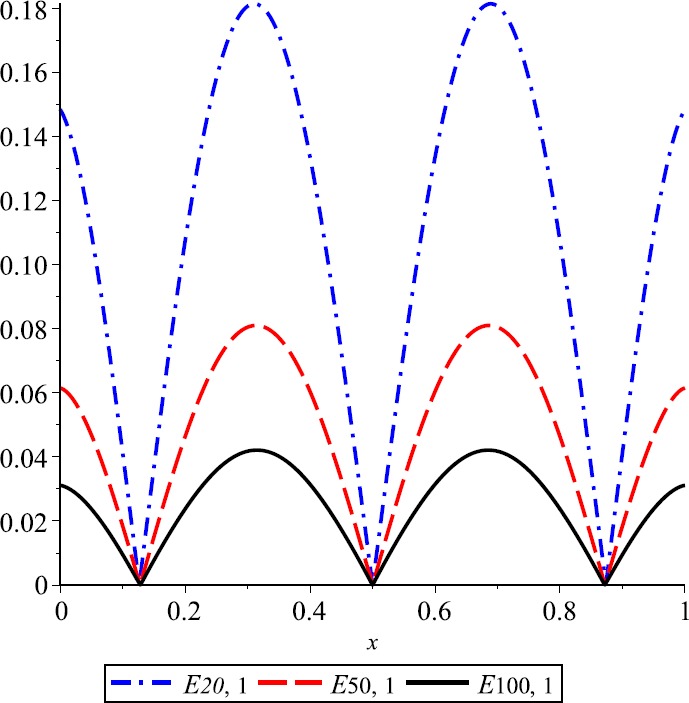
Error of approximation

For $\lambda=1$, the convergence of *λ*-Kantorovich operators to $f(x)=\operatorname{sin}(2\pi x)$ is illustrated in Fig. 3. Also, for $n=20,50,100$, the error functions $E_{n,\lambda}$ are given in Fig. 4.

### Example 3

**Figure 5:**
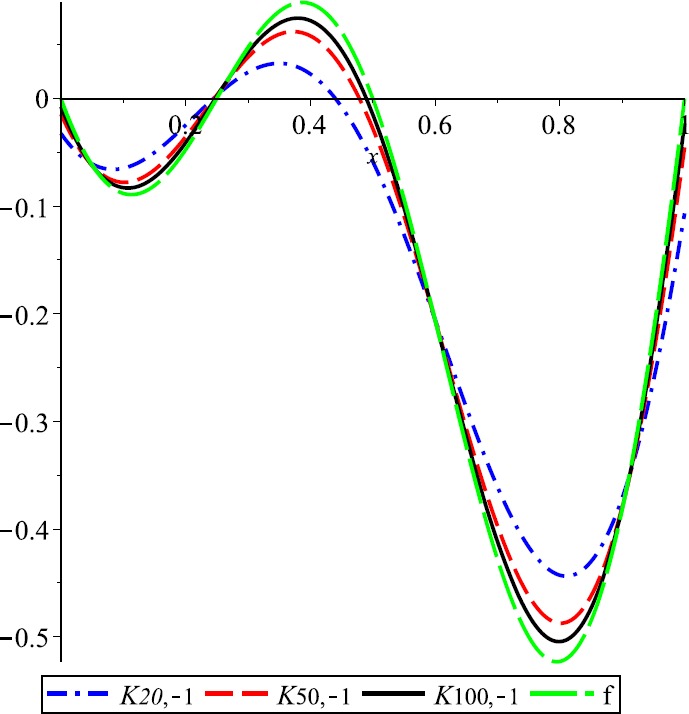
Approximation process

**Figure 6:**
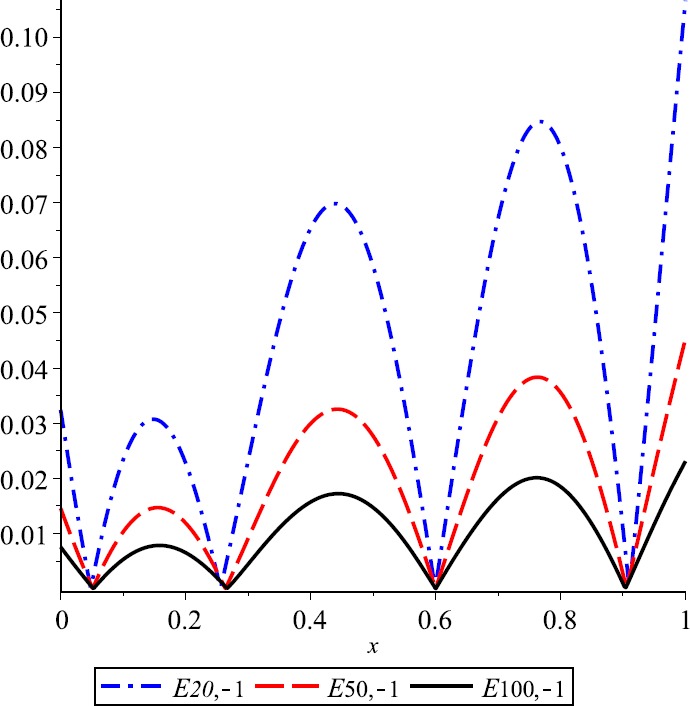
Error of approximation

For $\lambda=-1$, the convergence of *λ*-Kantorovich operators to $f(x)= (x-\frac{1}{4} )\operatorname{sin}(2\pi x)$ is illustrated in Fig. 5. Also, for $n=20,50,100$, the error functions $E_{n,\lambda}$ are given in Fig. 6.

### Example 4

**Figure 7:**
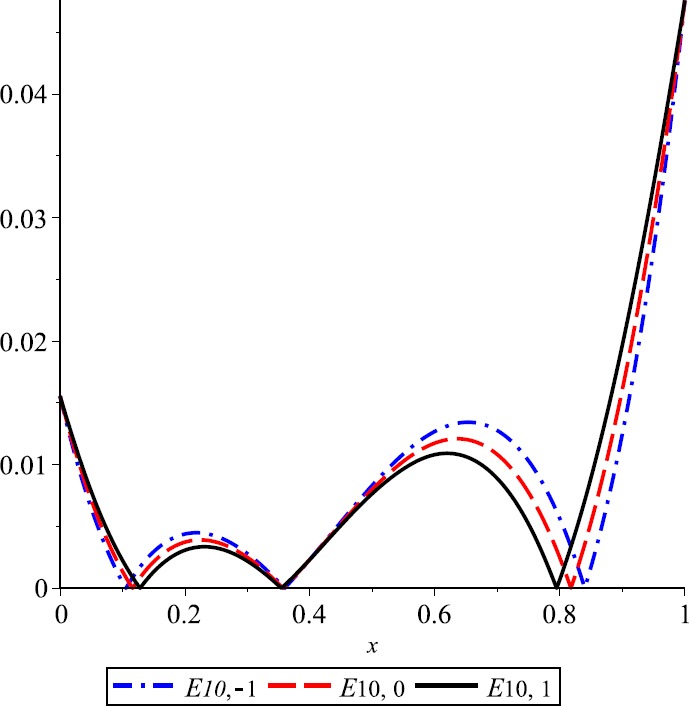
Error of approximation

Let $f(x)= (x-\frac{1}{4} ) (x-\frac {1}{2} ) (x-\frac{3}{8} )$ and $n=10$. In Fig. 7, we give the graphs of error functions for $\lambda=-1,0,1$. We can see that in this special case the error for *λ*-Kantorovich operators $K_{10,\lambda}, \lambda=-1,1$, is smaller than for $K_{10,0}$, that is the classical Kantorovich operator.

## Conclusion

The classical Kantorovich operators are the integral modification of Bernstein operators so as to approximate Riemann integrable functions defined on the interval $[0,1]$. Using the Bernstein operators depending on the parameter *λ* introduced by Cai et al. [11], in this paper we considered a new generalization of Kantorovich operators that improves in certain cases the rate of convergence of the classical ones. A lot of numerical examples were considered in this paper in order to show the relevance of the results.
